# ABO-Incompatibility: Time to Challenge the Paradigm of Equivalence in Live-Donor Kidney Transplantation?

**DOI:** 10.3389/ti.2022.10281

**Published:** 2022-02-08

**Authors:** Farsad Eskandary, Georg A. Böhmig

**Affiliations:** Department of Nephrology and Dialysis, Division of Medicine III, Medical University of Vienna, Vienna, Austria

**Keywords:** ABO-incompatible kidney transplantation, living donation, graft survival, induction, propensity score

Over the past decades, ABO blood group-incompatible live-donor kidney transplantation (ABOi-LDKT) has evolved significantly. Initial reports of—sometimes even inadvertent—crossing of non-permissive ABO blood group barriers have led to a broad use of such transplants as part of the clinical routine on numerous transplant units (1). In 2001, antigen-specific immunoadsorption, a highly efficient method for selective anti-AB antibody depletion was introduced by Tydén et al. (2) and this has led to the set-up of successful ABOi transplant programs in many European countries. Another major improvement was the use of CD20 antibody rituximab; this replaced splenectomy, which sometimes put recipients at significant risk of bleeding (3). Reports of their mid-to long-term results, accompanied by those from two other innovative European transplant centres, suggested excellent results with respect to patient- and graft survival rates, and noted that these were comparable with ABO-compatible live-donor kidney transplantation (ABOc-LDKT) (4–6). Since then, several large meta-analyses and registry studies have reported differing results regarding the equivalence of ABOi- and ABOc-LDKT. These have included information regarding the choice of the ideal induction regimen, as well as the decision to preferentially refer such donor/recipient pairs to national or international kidney paired exchange programs (7-10).

In this issue of Transplant International, de Weerd et al. (11) analyzed a large and well-characterized multicentric cohort of ABOi-LDKT from six different Dutch transplant centres, spanning a period of 14 years. They applied propensity score matching and used cause-specific Cox models to compare ABOi-LDKT with ABOc-LDKT and ABO-compatible deceased-donor (ABOc-DDKT) transplant outcomes.

A key finding was that patient survival was comparable between ABOi-LDKT and ABOc-LDKT, but was better than ABOc-DDKT. However, when looking at death-censored graft survival, ABOi-LDKT was associated with a higher risk for allograft loss, with a hazard ratio of 2.63 [95% CI: 1.72–4.01] when compared to ABOc-LDKT, and revealed results comparable with ABOc-DDKT. The authors applied a well-developed causal model to detect associations between potential confounding variables that they adjusted for in their final model.

The increased risk for graft loss in ABOi-LDKT versus ABOc-LDKT still remained, even when applying sensitivity analysis where dialysis duration prior to transplantation, diabetic nephropathy and use of rituximab as induction agent were excluded. Interestingly, inclusion of dialysis duration prior to transplantation in the model reduced differences regarding the observed benefit in patient survival between ABOi-LDKT versus ABOc-DDKT. One may argue that the patient-mortality benefit of ABOi-LDKT versus ABOc-DDKT observed in the other models could at least in part be explained by the fact that ABOc-DDKT patients had spent—not unexpectedly—a longer time on dialysis when compared to ABOi-LDKT (median 1,152 versus 216 days, respectively).

When comparing this study to a published cohort of equal granularity, the findings are, to an extent, in contrast to an analysis of >100 ABOi-LDKT performed in Freiburg, Germany (12). In this monocentric cohort study, Langhorst et al. (12) found no differences in patient mortality and graft survival rates when compared to those recorded for a well-matched cohort of ABOc-LDKT.

Looking at rejection rates after ABOi-LDKT, the study by de Weerd et al. (11) did reveal somewhat higher numbers compared to the study from Freiburg (Overall rejection rate 29% vs. 25%, respectively). A strength of their study is the additional reporting of recipient blood groups, as blood group O is—as has been pointed out by the authors—overrepresented in the Dutch population (about 66% of ABOi-LDKT recipients in their study had blood group O). Recipients with blood group O were shown to be associated with higher anti-AB antibody levels, which may account for the reported high rejection rates (13). As mentioned by the authors, reporting of recipient blood groups should be a pre-requisite in publications about ABOi-LDKT.

One aspect of the study by de Weerd et al. (11) which merits further discussion is the lack of data regarding calcineurin-inhibitor (CNI) levels. These might have some impact on outcomes in the immunologically demanding setting of ABOi-LDKT versus ABOc-LDKT. Although CNI trough level goals were reported, the earlier trough level goals of 10–15 ng/ml were later lowered to 8–12 ng/ml after introducing basiliximab or alemtuzumab as induction agents, but were set identical in both LDKT cohorts. It would be interesting to see the median CNI trough-level corridor achieved by clinicians in the six centres. This information might help increase clinicians’ confidence when working with ABOi-LDKT recipients. The knowledge that target-CNI goals were mostly achieved in the present study could then also be interpreted in the context of pre-transplant anti-AB IgG and IgM titer subgroups, as higher titers were associated with higher rejection frequency (11).

Lastly, the study sheds light on the effects of different induction regimens used in ABOi-LDKT. Their study clearly shows that sole use of rituximab was associated with a much higher rejection rate compared to combined rituximab/basiliximab or alemtuzumab treatment. Although use of rituximab only was also reported in the early studies by Tydén et al. (2) , most centres in the study by de Weerd et al. (11) included additional basiliximab in their later protocols. This was also the case in centres not discussed in the study. Their findings however, strongly support the use of combined rituximab/basiliximab or—if available—alemtuzumab as induction agents in ABOi-LDKT. The question whether pre-transplant antibody depletion and rituximab is necessary in all patients prepared for ABOi-LDKT is, however, still up for discussion, since the study by Masterson et al. (14) from Melbourne, Australia has shown favorable results in the presence of low anti-AB antibody titers where rituximab and immunoadsorption were omitted.

In conclusion, the study by de Weerd et al. (11) does confirm the suspicion that ABOi-LDKT might not be as beneficial as ABOc-LDKT, but still shows a clear benefit over ABOc-DDKT. If ABOi donor/recipient pairs do have the option to enter kidney-paired donation programs and receive an ABOc-LDKT, this might be a better alternative with respect to long-term graft survival, but there is a need for careful evaluation and counseling by the treating physicians on an individual level.
